# AntAngioCOOL: computational detection of anti-angiogenic peptides

**DOI:** 10.1186/s12967-019-1813-7

**Published:** 2019-03-04

**Authors:** Javad Zahiri, Babak Khorsand, Ali Akbar Yousefi, Mohammadjavad Kargar, Ramin Shirali Hossein Zade, Ghasem Mahdevar

**Affiliations:** 1 Bioinformatics and Computational Omics. Lab (BioCOOL), Department of Biophysics, Faculty of Biological Sciences, Tarbiat Modares University (TMU), Tehran, Iran; 2 Computer Engineering Department, Faculty of Engineering, Ferdowsi University of Mashhad, Mashhad, Iran; 3 Department of Computer Engineering, Faculty of Engineering, University of Science and Culture, Tehran, Iran; 4 Computer Engineering Department, Sharif University of Technology, Tehran, Iran; 5 Department of Mathematics, Faculty of Sciences, University of Isfahan, Isfahan, Iran

**Keywords:** Machine learning, Angiogenesis, Anti-angiogenic, Peptide, Cancer, Cancer treatment

## Abstract

**Background:**

Angiogenesis inhibition research is a cutting edge area in angiogenesis-dependent disease therapy, especially in cancer therapy. Recently, studies on anti-angiogenic peptides have provided promising results in the field of cancer treatment.

**Methods:**

A non-redundant dataset of 135 anti-angiogenic peptides (positive instances) and 135 non anti-angiogenic peptides (negative instances) was used in this study. Also, 20% of each class were selected to construct an independent test dataset (see Additional files 1, 2). We proposed an effective machine learning based R package (AntAngioCOOL) to predict anti-angiogenic peptides. We have examined more than 200 different classifiers to build an efficient predictor. Also, more than 17,000 features were extracted to encode the peptides.

**Results:**

Finally, more than 2000 informative features were selected to train the classifiers for detecting anti-angiogenic peptides. AntAngioCOOL includes three different models that can be selected by the user for different purposes; it is the most sensitive, most specific and most accurate. According to the obtained results AntAngioCOOL can effectively suggest anti-angiogenic peptides; this tool achieved sensitivity of 88%, specificity of 77% and accuracy of 75% on the independent test set. AntAngioCOOL can be accessed at https://cran.r-project.org/.

**Conclusions:**

Only 2% of the extracted descriptors were used to build the predictor models. The results revealed that physico-chemical profile is the most important feature type in predicting anti-angiogenic peptides. Also, atomic profile and PseAAC are the other important features.

**Electronic supplementary material:**

The online version of this article (10.1186/s12967-019-1813-7) contains supplementary material, which is available to authorized users.

## Background

Angiogenesis is the process of formation of new blood vessels from pre-existing vessels to make a supply of nutrients and a waste disposal pathway [1]. Angiogenesis is a normal and fundamental physiological process in growth and development [2–4]. However, it is a vital event in cancer progression—transition of tumor from a benign state to a malignant one—and spread of a tumor (metastasis) [5–7]. Nowadays, decreasing or inhibiting angiogenesis is a cutting edge research area in cancer therapy which also plays a key role in other angiogenesis-dependent disease therapy [1, 8–15].

Besides other therapeutic peptides, recognition of the anti-angiogenic peptides has stimulated great interest among researchers in the cancer treatment field during recent years [16–23]. However, there are very rare studies in computational detection of ant-angiogenic peptides [8].

In this paper, we have proposed an efficient machine learning based R package to detect anti-angiogenic peptides, namely AntAngioCOOL. Five types of features have been used to encode peptides in order to predict anti-angiogenic ones. According to the obtained results, AntAngioCOOL reached to a satisfactory performance in anti-angiogenic peptide prediction on a benchmark non-redundant independent test dataset.

## Methods

### Dataset

We have used the gold standard dataset that has been recently published [8]. After removing redundant peptides, this dataset contained 135 anti-angiogenic peptides (positive instances) and 135 non anti-angiogenic peptides (negative instances). Also, a 20% of each class was selected to construct an independent test dataset (see Additional file 1).

### Features

The following subsections provide a brief description for each peptide feature. Moreover, Table 1 demonstrates the distribution of the features that have been used to encode each peptide.

**Table 1:** Distribution of the features used to encode each peptide

Feature type	No. of features
PseAAC (λ = 6)	28
k-mer composition	
k = 2	400
k = 3	8000
k = 4	160,000
k-mer composition (reduced alphabet^a^)	
k = 2	64
k = 3	512
k = 4	4096
Physico-chemical profile	1910
Atomic profile	80
Total	175,062

^a^To compute k-mer composition features, the reduced amino acid alphabet proposed by Zahiri et al. was applied: the 20 alphabet of amino acids was reduced to a new alphabet with size 8 according to 544 physicochemical and biochemical indices that extracted from AAIndex database (C_1_ = {A, E}, C_2_ = {I, L, F, M, V}, C_3_ = {N, D, T, S}, C_4_ = {G}, C_5_ = {P}, C_6_ = {R, K, Q, H}, C_7_ = {Y, W}, C_8_ = {C}). We computed k-mer composition for k = 2, 3, 4 for each peptide

### Pseudo amino acid composition

We used pseudo amino acid composition (PseAAC) which has been used effectively in predicting cell penetrating peptides [21]. Unlike the simple amino acid composition, PseAAC considers the sequence-order information of the peptide. Interested readers may refer to [24] for further information on PseAAC.

### k-mer composition

k-mer composition shows the fraction of all possible subsequences with length *k* in the given peptide. Also, the reduced amino acid alphabet proposed by Zahiri et al. [25] has been applied to compute another k-mer composition: the 20 alphabet of amino acids have been reduced to a new alphabet with size 8 according to 544 physicochemical and biochemical indices extracted from AAIndex database [26] (C_1_ = {A, E}, C_2_ = {I, L, F, M, V}, C_3_ = {N, D, T, S}, C_4_ = {G}, C_5_ = {P}, C_6_ = {R, K, Q, H}, C_7_ = {Y, W}, C_8_ = {C}). We have computed k-mer compositions for k = 2, 3, 4 for each peptide.

### Physico-chemical profile

In order to compute this feature type, 544 different physico-chemical indices were extracted from AAIndex [26]. To remove redundancies, a subset of indices with correlation coefficient less than 0.8 and greater than − 0.8 were selected, which resulted in 191 non-redundant physico-chemical indices.

This feature type has been extracted for 5 amino acids of N-termini (5-NT) and C-termini (5-CT). Finally, each peptide has been encoded as a 10 × 191-dimensional feature vector as below:

$$\left( {PC_{1}^{1} ,PCP_{2}^{1} , \ldots ,PCP_{191}^{1} , \ldots ,PC_{1}^{10} ,PCP_{2}^{10} , \ldots ,PCP_{191}^{10} } \right)$$

where $PC_{j}^{i}$ is the value of the *j*th physico-chemical index for the *i*th amino acid of the peptide (for *i *= $1, \ldots ,5$ in the 5-CT and *i *= $6, \ldots ,10$ in 5-NT)

### Atomic profile

A 50-dimensional feature vector has been used to encode each peptide according to its atomic properties as below:

$$\left( {AC_{1}^{1} ,AC_{2}^{1} , \ldots ,AC_{5}^{1} , \ldots ,AC_{1}^{10} ,AC_{2}^{10} , \ldots ,AC_{5}^{10} } \right)$$

where $AC_{1}^{i}$ through $AC_{5}^{i}$ represent the frequency of five types of atoms: C, H, N, O, S in the *i*th amino acid of the peptides (for *i *= $1, \ldots ,5$ in the 5-CT and *i *= $6, \ldots ,10$ in 5-NT). For details of atomic composition for each 20 natural amino acid see [17].

### Machine learning method

**Fig. 1:**
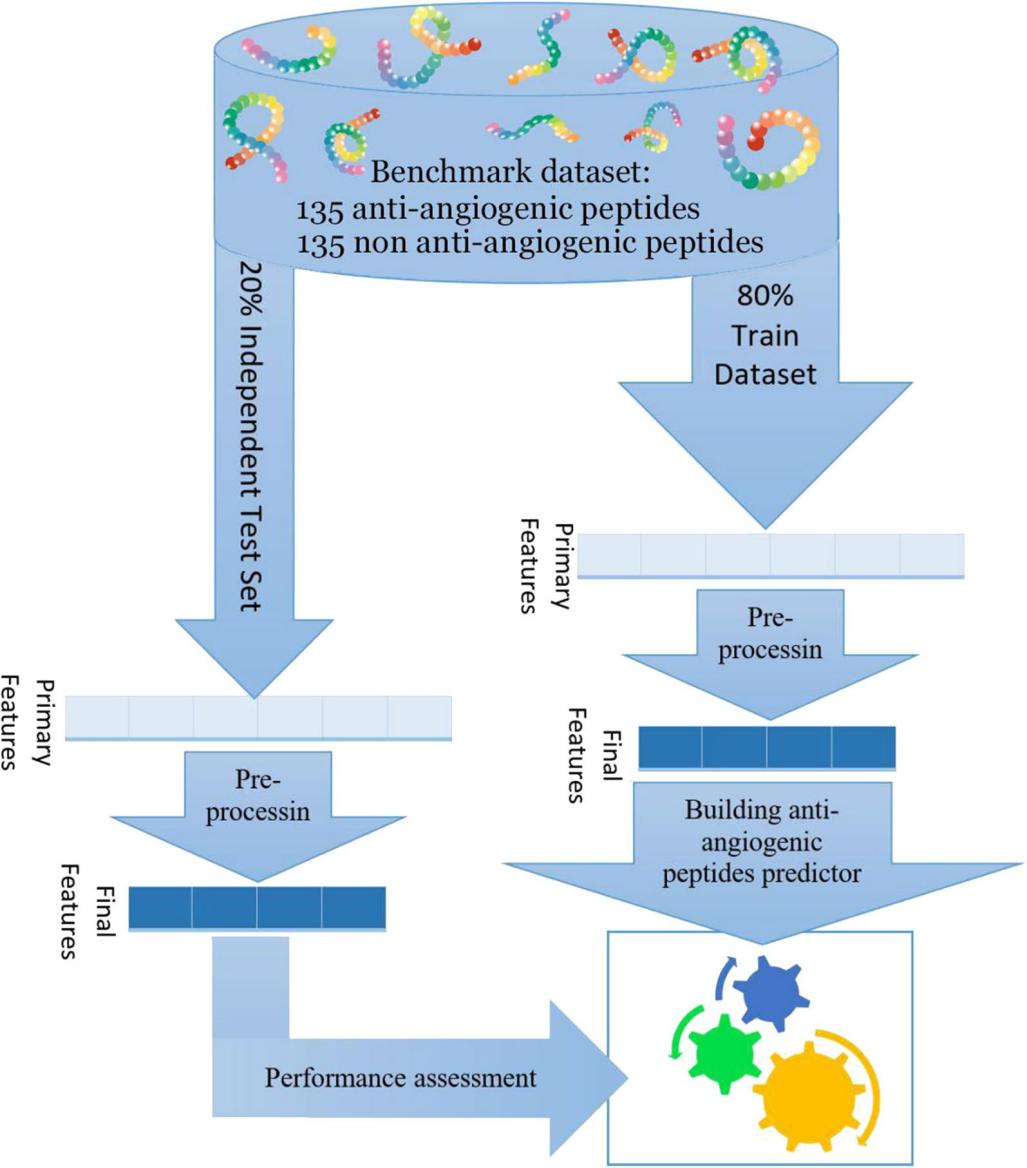
Schematic representation of the proposed method (AntAngioCOOL) for anti-angiogenic peptide prediction

To build a powerful anti-angiogenic peptide predictor, 227 different classifiers (see Additional file 1) in the caret package [27] were examined. Finally, the three best classifiers (those with best sensitivity, specificity and accuracy) were selected to be included in the AntAngioCOOL package. Figure 1 provides a schematic representation of the proposed method.

### Evaluation parameters for the prediction performance

The training dataset was used to train the classifier, and then the classifier was evaluated using the test data. The predictions made for the test instances were used to compute the following performance measures:

$$Sensitivity = \frac{TP}{TP + FN}$$

$$Specificity = \frac{TN}{TN + FP}$$

$$Accuracy = \frac{TP + TN}{TP + FP + TN + FN}$$

where, TP and TN are the number of correctly predicted anti-angiogenic peptides and non anti-angiogenic peptides, respectively. Similarly, FP and FN are the number of non anti-angiogenic peptides and anti-angiogenic peptides wrongly predicted as anti-angiogenic peptides and non anti-angiogenic peptides, respectively.

## Results

### Preprocessing

To remove non-informative features, which can lead to reducing the computational cost without losing the prediction performance, *nearZeroVar* function from caret package [27] was utilized. This function eliminates those features that have one unique value (i.e. are zero variance features) or features with both of the following characteristics: they have very few unique values relative to the number of samples and the ratio of the frequency of the most common value to the frequency of the second most common value is large. *nearZeroVar* was applied to the extracted features using its default parameters. Interestingly, less than 2% of the extracted features (2343 out of 175,062) were selected as informative ones to construct the prediction models (see Additional file 1 for more details).

### Prediction performance

**Fig. 2:**
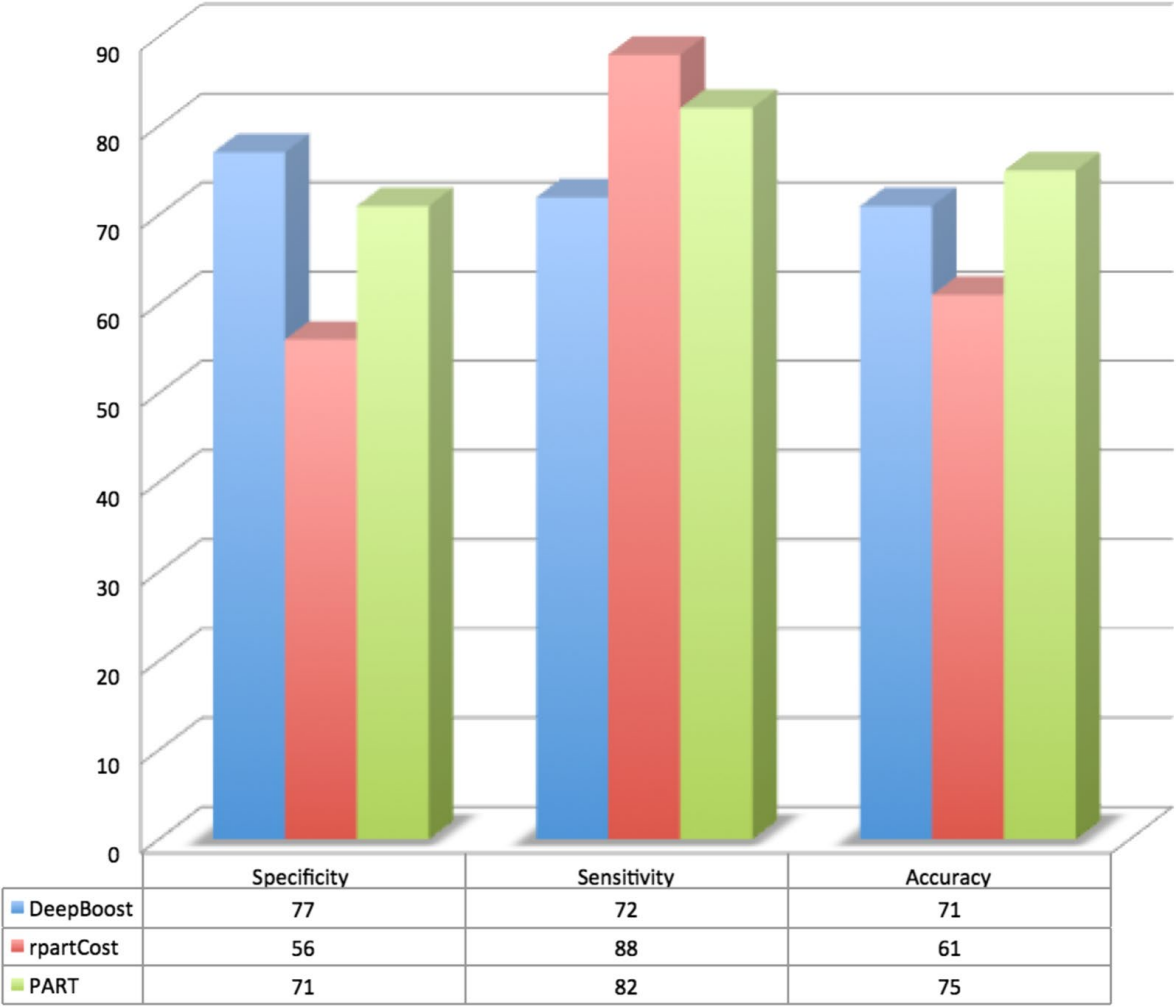
Prediction performance of the three selected classifiers among 227 classifiers to be included in AntAngioCOOL package in the test dataset

The performance results of the 227 classifiers with accuracy > 50% in the independent test set have been shown in Additional file 1: Figures S1–S3. We have selected the three best classifiers to be included in the AntAngioCOOL package (Fig. 2): the most sensitive classifier (rpartCost with 88% sensitivity), the most accurate classifier (PART with 75% accuracy) and the classifier with the highest specificity (DeepBoost with 77% specificity). Availability of these three classifiers can help biologists with different questions in mind; e.g. having a list of candidate peptides, what is the narrow list of confident anti-angiogenic peptides or what is the more extended sub-list of candidate anti-angiogenic peptides that contains almost real anti-angiogenic peptides.

## Discussion

### Physico-chemical profile is the most important feature type

**Fig. 3:**
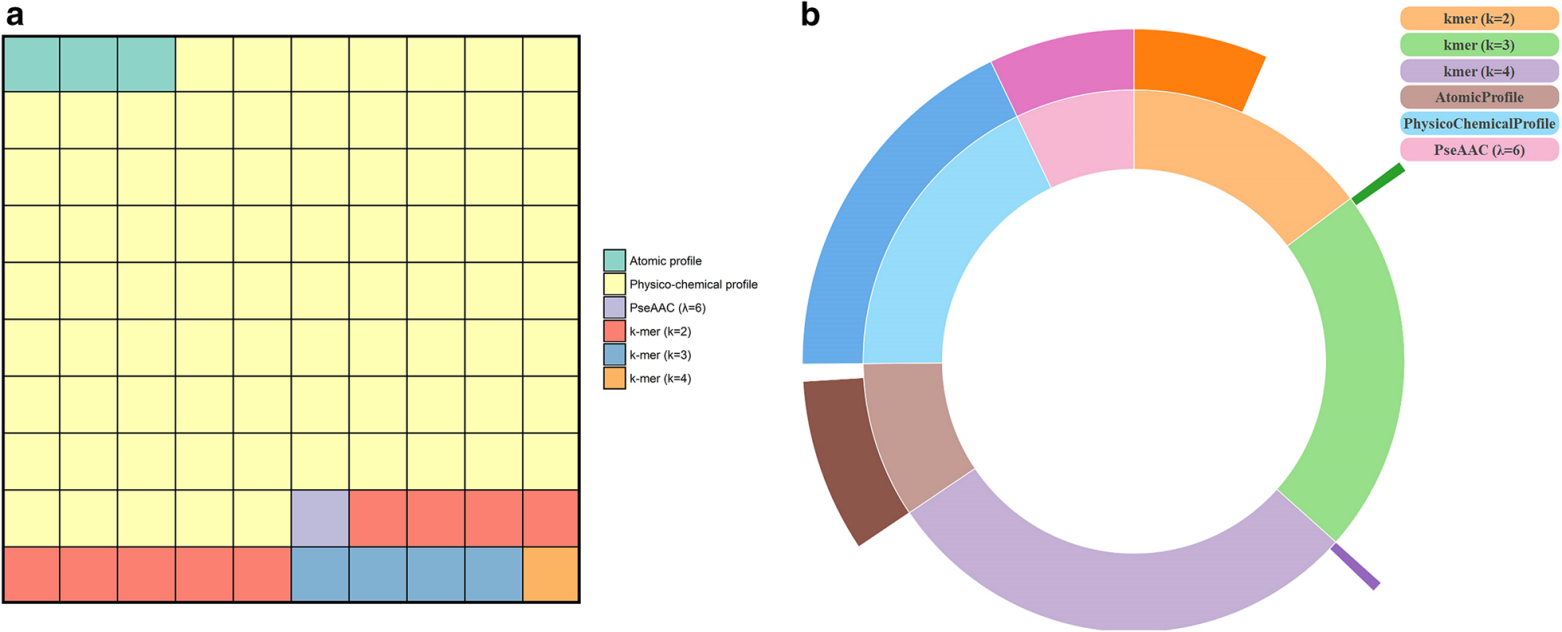
Feature importance according to their contribution in final selected features (informative features). **a** Square chart of the distribution of different feature types in the final important features. **b** Inner layer of this sunburst chart shows the distribution of different feature types in the primary extracted features (in log scale). The outer layer shows the proportion of each feature type selected as informative features

Physico-chemical profile is the main feature type in the final selected set of features which is 82% of the final features (Fig. 3a). Interestingly, almost all physico-chemical profile features were selected (1909 out of 1910). Dipeptide and tripeptide compositions are the other important feature types that comprise 9% (200 features) and 4% (101 features) of the final features, respectively. Moreover, Fig. 3b shows the percentage of each feature type that was selected as a subset of the final features.

### Sequence-order information is useful for anti-angiogenic peptide prediction

As the Fig. 3b shows, in addition to the physico-chemical profile, a considerable percentage of the atomic profile (91.3%) and all the PseAAC features were selected as informative features (Additional file 2 for more details). One of the important common aspects of these three feature types is that they take the sequence-order information of the peptide into account. Therefore, this results stress out that the sequence-order information is an effective factor in anti-angiogenic peptide prediction.

### Dipeptide is the most important feature among k-mer composition features

One of the interesting obtained results is that 43.1% of dipeptides were selected as informative features while for tripeptides and quadpeptides there are very small number of informative features for predicting anti-angiogenic peptides: 101 out of 8512 (1.2%) and 32 out of 164,096 (0.02%), respectively. So, dipeptide composition is the most important k-mer composition in anti-angiogenic peptide prediction.

### Comparison with the current state-of-the-art methods

The proposed method has been trained and tested with the same data used for AntiAngioPred [8]. Results reveal that AntAngioCOOL has a higher accuracy (77% vs. 75%) and considerable higher sensitivity (88% vs. 65%). Therefore, AntAngioCOOL package can be used more effectively in anti-angiogenic peptide prediction, especially when one is interested in detecting almost anti-angiogenic peptides (in the cost of having some false positives) in a given list of peptides.

## Conclusion

In this study an R package (AntAngioCOOL) was proposed to predict anti-angiogenic peptides. AntAngioCOOL exploits five descriptor types for a peptide of interest to perform the prediction including: PseAAC, k-mer composition, k-mer composition (reduced alphabet), physico-chemical profile and atomic profile. After removing the non-informative descriptors, only 2% of the extracted descriptors were used to build the predictor models. AntAngioCOOL includes three different models that can be selected by the user.

The results disclosed that physico-chemical profile is the most important feature type. Also, atomic profile and PseAAC are the other important features. Therefore, it can be concluded that sequence-order information plays a critical role in anti-angiogenic peptide prediction. In addition, according to the results dipeptide has the most contribution in anti-angiogenic peptide prediction among the k-mer composition features.

## Additional files

**Additional file 1.** Supplementary Materials and Methods. Train and test datasets; 227 different classifiers.

**Additional file 2.** Supplementary Results. Results of feature selection and feature importance analysis.

## Abbreviation

PseAAC: pseudo amino acid composition
