# Supplementary material for: AntAngioCOOL: computational detection of anti-angiogenic peptides
Source: J Transl Med. 2019 Mar 4;17:71. doi: 10.1186/s12967-019-1813-7 (PMC6399940; doi:10.1186/s12967-019-1813-7)
Supplement: Supplementary file 1 — Additional file 1. Supplementary Materials and Methods. Train and test datasets; 227 different classifiers. [file 12967_2019_1813_MOESM1_ESM.docx]

**Train dataset:**

**POSITIVE DATASET (107 sequences)**

AAPFLECQGRQGTCHFFAN

ANIKLSVQMKLFKRHLKWKIIVKLNDGRELSLDA

CDSDSDITWDQLWDLMK

CETWRTETTGATGQASSLLSGRLLEQKAASCHNSYIVLCIENSFMTSFSK

CKITRCPMIPCYISSPDECLWMDWVTEKNINGHQAKFFACIKRSDGSCAWYRGAAPPKQEFLDIEDP

CQNHHAKHGKVC

DDDDDNDKIPDDRDN

DDDDKRAGSPSGGPFCALARQPLTGSPPNERAFFCSSRDV

DGRELCLDPKENWVQRVVEKFLK

DGRKICLDPDAPRIKKIVQKKL

DLWIRETLTSPKSLTG

DPFFKVPVNKLAAAVSNFGYDLYRVRSSTSPTTN

DPPEGLUGTKPPROH

DRSTREPIYMSTI

DSSPVSTEQLAPTA

EGLPGPQGPKGFPGLPGLTG

EIPSCESSASPDQSDSSVPPEE

EKSSRPEFYKVILGAHEEYIRG

EKYEGKISKTMSGLDCQAWDS

ESLARPCAPGAPAEARL

FCNINNVCNFASRNDYSYW

FLKDHRISTFKNWPF

FLSSRLQDLYSIVRRADRAA

GFHDHGPCDPPSHK

GHRATSDLASTGEESQD

GPWEDCSVSCGGGEQLRSR

GPWEPCSVTCSKGTRTRRR

GPWERCTAQCGGGIQARRR

GPWGDCSRTCGGGVQFSSR

GPWGPCSGSCGPGRRLRRR

HGLGHGHEQQHGLGHGHKFKLDDDLEHQGGHVLD

HGSTTLRDITV

HHPHGHHPHGHHPHGHHPHG

HKLINTEGHHS

HTHQDFQPVLHLVALNTPLSGGMRGIR

IMRIKQGQIGQMTI

INEFLERSGIPRQRNQ

INGSLDKRLLPDVET

INGSLDKRVQDCYHG

INLEACLKRGRT

ITMQGIQGQKIRMIMF

KAFDITYVRLKF

KCGHKHQCAVHN

KIKSCYYLPCFVTS

KRFKQDGGWSHWSPWSSCSVTCGDGVITRIRLCNSPSPQMNGKPCEGEARETKACKKDACPI

KSVRGKGKGQKRKRKKSRYK

LHCPALVTYNTDTFESMPNPEGRYTFGASCV

LLRISLLLIQSWLE

LPGLTGSKGVRGISGLPGFSG

LRRFSTMPFMFCNINNVCNF

LRSRGELVAKFLAGEQSPEDYVAE

LSSTCILVLVKDILVLVVKEILVLVVKDKPI

LVPLPKIKNSTFT

LVPRGSRAGSPSGGPFCALARQPLTGARLMSGLFFALHET

MEPECNLNCTD

MFSPILSLEIILALATLQSVFAQPVICTTVGSAAEGS

MLQNSAVLLLLVISASA

NGKQVCLDPEAPFLKKVIQKILDS

NGREACLDPEAPMVQKIVQKMLKG

NGRKACLNPASPIVKKIIEKMLNS

NVLLSPLSVATALSALSLGAEQRTES

PGLKGKRGDSGSPATWTTRG

PTGERLRTCERLSYP

QEPHRHSIFTPQTNPRADLEKN

QMIVIELGTNPLKSSGIENGAFQGMK

QPWGTCSESCGKGTQTRAR

QPWSQCSATCGDGVRERRR

RCRLAERRQIAK

RIFGESVSLRVQDWEW

RPFVEMYSEIPE

RQVFQVAYIIIKA

RRPAAAGKRRREKQRPSDKPRR

RRPKGRAMRREKQRPSDKPRR

RRPKGRGKRRREKQRPTDCHLCGDAVPRR

SAWRACSVTCGKGIQKRSR

SEWSDCSVTCGKGMRTRQR

SKRKSRPVSVKTFEDIPLEEP

SKWSECSRTCGGGVKFQER

SPNITVTLKKFPL

SPSTHPNEGLEENYCRNPDN

SPWSKCSAACGQTGVQTRTR

SPWSPCSGNCSTGKQQRTR

SPWSPCSTSCGLGVSTRI

SPWSQCTASCGGGVQTR

SPWTKCSATCGGGHYMRTR

SRTVRKTSRLWSSLSLNTCNNVHSKS

SSTSPHRPRFS

TAWGPCSTTCGLGMATRV

TEWSACNVRCGRGWQKRSR

TEWSVCNSRCGRGYQKRTR

TEWTACSKSCGMGFSTRV

TGASSEEEDPF

TKPPRKRPPKTKKRPPKTTKPPRGZOG

TKWTPCSRTCGMGISNRV

TLPFAYCNIHQVCHYAQRNDRSYWL

TQWTSCSKTCNSGTQSRHR

TSLDASIIWAMMQN

TSWSQCSKTCGTGISTRV

TTITGKKCQSWAAMFPHRHSKT

VGSGGCMFGNGK

VIFEWTLLQVLSESDQDQSLEVFLT

VVGSPSAQDEASPL

WTRCSSSCGRGVSVRSR

YCNINEVCHYARRNDKSYWL

YPYDVPDYASL

YRIPIVRRLQRR

YTMNPRKLFDY

**NEGATIVE DATASET (107 sequences)**

ADNWQSFDRWKDH

AEALAALRALADKNQVF

AFAQFGSDLDAATQKLLNRGARLTELMKQPQ

AGAGYALLALIGTEAAS

AKAAETKSSSEQELRITQS

ATSINNSSLPDV

AVVQKRFGFPEGSV

DDVWNMKYLRGFKWADLMEQVQRE

DKAFIAFLEETFDQFLP

DMEAFTKLTDNIFLE

DVSKLKEGEQYMSFCTFPGHSALM

EALDAARYYANV

EASGPSFVSSHYLQESPGGISLEGSELTFPD

EDWSLDSRPGKSTKNSRNK

EGELLILENVRFNKGEKKDD

EHNDLRLCCKQIVEEA

EKAGLKIVAAKMLQLSQAQAEG

EKQIEQLVAQDLVRHFADLYRIDIPT

ELLIEDHIKTACNWGTTHK

ENGSTAIVVGRPITQAADPQKAYE

ERYAALLHDLGKAKTPSDILPRHHGHDLAGVEPVRKVNQRLRAPKHCAEL

FEPQVMKIMANVRPDRQTVLFSATFPRNMEALARKTLNKPVEIVVGGKSVVAPEITQIVEVR

FFTPSASHPAYVNFA

FGRLGTMFGSDLYNIKPDLV

FTSALSRAQKT

FTVRKISNGEGVERAFQTH

GALTDPTAQLVYLQKDGGL

GGPAERLTYEGDYNARGV

GHENISTTQIYTHLDFQHLADVYDQAHPRARKKSSQHKEE

GIVFQFFNLIPTLTVLENITLP

GKGVKTEFNRHVEDIKRESDGAWVL

GKSVADAIAILTFTPNKAAEII

GLHKGNKVNLTLRPAPANTGLIFRRVD

GMIFLELNFKGAEEIYYKHVHCRGGCSVFFSKISGVLTFM

GPEGMLSIAAPARDLKLATIELEHSHPLGRLWDIDVLTPEGEILSRRDYSLPPRRCLLCEQSAAVCA

GSGRTDARVHAQGQ

GSKFDSSLDRNRPFEFTLGAGQVIK

GTSIVGIVENGISVLGKIF

HGGRVTLMEITDDGLAILQFGGGCNGCSMVDFTL

HSGNIWVDSDPARKSNPRFIVLD

HSSREKIVIPFFSLLIKDIYFLNEGCA

HVLSRLSYISALGMMTRITS

HWMYQGKHVLIIFDD

IAELGTAEFPRLRIGIGRPAP

IEHPVLMARKPRFR

IEVTHWVQSRRAYAQGALEAARRLIGRPP

IFTFAGLIDHSHDFIIGFHAV

IHRAAGPALINACY

IIYITEEMGLLLGYSPIEILEKRF

INLTIAVHNGR

IVDDWIYMIEEICKI

IWIDPGFGFAKSVQQNTELLKGLDRVCQLGYPVL

KFGADCKYKFES

KLCGTNSDAYGFSANLDDS

KLLDIADLHSEMKPLH

KMVTADYIKEGA

KVSVWSKVLRSDAAWDDK

LASAYGLAKHRDGRWEWA

LGYLGPDLADSAIAVNESIIPKFLRLVDPTAAELQNF

LHRGRIPEHQREESEV

LLSEYIPSVPNCWSLLKNKKT

LMEYEQNENPMK

LVAPVTVGKGA

LVVVPPYVIRY

MASGNAVCGSSAIAAVEP

MQSLVDIAAVTELAHAAGAKV

MRIVDLGAAPGGWSQVAAKK

MTGLVKWFNPE

MVFITVSTGVGGGVVSGGKLLTGPGG

MVSSEKAMANPDSMEIDSQTISQQVLITSQSGSV

MYNSLLRMTGACHKKCVPPH

NDNTPEILYPTI

NGWLHCPADPDLIF

NITVMTSGFAFHYYVNNPH

NTKFDELMEFP

PDLCSWEEAQLSS

PKLTALVENVAEQQGINLTS

QAGADISMIGQFGVGFYSA

QAITDIHLDRV

QAQQKIILETFILFEDEVGKKL

QGCKMNNINVVYTPWANLKK

QMLEEGLLDEVQALLAAGIKGN

QTTIHVLPTAPTTVNVT

RELAAEVGSLLT

RHPDCKIVRRRGRV

RPGTPLFTVKAYL

RSERLAKLNQILRI

RVEQPENPMLDARVQAFRIA

SGNMLAGGGTLYLYALGMG

SMGPMPESGQLVFQTANLT

STDVSWEELRDTE

SYDLGERKPSSAAYQKAPT

SYRDKEMSATFRQIL

TEGIDAMGEVTIRLRRDGQLFSGHAA

TIASMPAVDEINRLSN

TLPHQRLIVATDRGIFYKM

VAATDGVGTKLKIAIDTGN

VAFKPNSTNIHVENVTVYG

VCHGNCPQSNNAFFQPLDP

VFSTTSLVVVAHYKGLTVA

VIVCLLGTAGLFLPPWLA

VKVIEAVRARTPKTT

VQDFGTALKVPK

VVRLAREPGKRESRYMH

YEDLRDESLKGLVDIGF

YFLIQSVSSTVMLLNGLYIFVN

YNLSDTIKAFSILLLTDLCI

**Tests Data**

**POSITIVE INDEPENDENT DATASET (28 sequences)**

ARPAKAAATQKKVERKAPDA

ASWSACSVSCGGGARQRTR

ATPFIECSGARGTCHYFAN

CELDENNTPMC

DFKLFAVYIKYR

DTAVTGLASPLSTGKILDQKAYSCANRLIVLCIENSFMTDARK

EDMNQKLFDLRGKFKRPPLRRVRMSADAML

GDVIDTDRDIDR

GVDITVIRPNH

GYCSWYRGWAPPDKSIINATDP

HNRTPENFPCKNL

INLEACLGRTLMD

IYSFDGRDIMTDPSWPQKVIWHGSSPHGVRLVDNYCEAWRTA

KNECLWTDMLSNFGYPGYQSKHYACIRQKG

MPTWAWWLFLVLLLALWAPARG

NGRKISLDLRAPLYKKIIKKLLES

QQMNQKDFLSLIVS

QRTESIIHRALYYDLIS

RGFTKMPHVQIHTEASESL

SAPFIECHGRGTCNYYANS

SPWDIASVTAGGVQKRS

SPWSQCSVRCGRGQRSRQVR

SPWSSASVTAGDGVDITRIR

SVSGGGHHHHHHGGG

TEENRELVSELKRP

TMPFLFCNVNDCNFASRNDYSYWL

TSWSPCSASCGGGHYQRTR

WDLVVVSAGVAEVGV

**NEGATIVE INDEPENDENT DATASET (28 sequences)**

AERWREAAKLI

ALVIGVIYATSMIFQSTSLV

AVYLFYGTKDCL

CSRDNKHTLHRE

DIAPDTLENLISEFVLREG

DLDDESIQGKLNFENFSLL

ENAKNRLGLAQAD

EYIDGSVIAQL

GRLKGEELAQYNLWLDYLDALE

ICRDIDLVRKLIKQAGLSLLAVERQENFPD

KEVDAKYIETKRSIVQHITQIPYY

KQVKDKVPDGVFIFLTPPDLAELKSRIIGR

KTSTEAGVNLVVG

PDEVTIGIVRERLG

PLIVLKDSIGREVINRSLIRVR

QGGAQRGGFTGPIP

QLPLQQQQQQQQQQQQQQQ

RAGSKRWLGKRPVVRGVVMNPVDHPHGGGEGRAPIGRKKPTTP

RFRVLPQGLKVKQVEREDAGVYVCKATNGFGSLSVNYTLVVL

RKEAKRRYNEGALPGFDPA

RLQLGKMLNLIDESKFA

RVIVVFHCEFSSERGPRMCR

TVEIVMGLEEEFQISVE

VCGTIYVGGKEVNQCMDKTSDNAI

VMFAALIFKKDTFFR

VQWILSFPRAPMGSVSVHV

VRYHYINKAYEVTMKIQIIS

YGEPGMQLFVYGREE

**List of examined classifiers**

1. ada
2. AdaBag
3. AdaBoost.M1
4. adaboost
5. amdai
6. ANFIS
7. avNNet
8. awnb
9. awtan
10. bag
11. bagEarth
12. bagEarthGCV
13. bagFDA
14. bagFDAGCV
15. bartMachine
16. bayesglm
17. bdk
18. binda
19. blackboost
20. blasso
21. blassoAveraged
22. Boruta
23. bridge
24. brnn
25. BstLm
26. bstSm
27. bstTree
28. C5.0
29. C5.0Cost
30. C5.0Rules
31. C5.0Tree
32. cforest
33. chaid
34. CSimca
35. ctree
36. ctree2
37. cubist
38. dda
39. deepboost
40. DENFIS
41. dnn
42. dwdLinear
43. dwdPoly
44. dwdRadial
45. earth
46. elm
47. enet
48. enpls.fs
49. enpls
50. evtree
51. extraTrees
52. fda
53. FH.GBML
54. FIR.DM
55. foba
56. FRBCS.CHI
57. FRBCS.W
58. FS.HGD
59. gam
60. gamboost
61. gamLoess
62. gamSpline
63. gaussprLinear
64. gaussprPoly
65. gaussprRadial
66. gbm
67. gcvEarth
68. GFS.FR.MOGUL
69. GFS.GCCL
70. GFS.LT.RS
71. GFS.THRIFT
72. glm
73. glmboost
74. glmnet
75. glmStepAIC
76. gpls
77. hda
78. hdda
79. hdrda
80. HYFIS
81. icr
82. J48
83. JRip
84. kernelpls
85. kknn
86. knn
87. krlsPoly
88. krlsRadial
89. lars
90. lars2
91. lasso
92. lda
93. lda2
94. leapBackward
95. leapForward
96. leapSeq
97. Linda
98. lm
99. lmStepAIC
100. LMT
101. loclda
102. logicBag
103. LogitBoost
104. logreg
105. lssvmLinear
106. lssvmPoly
107. lssvmRadial
108. lvq
109. M5
110. M5Rules
111. manb
112. mda
113. Mlda
114. mlp
115. mlpML
116. mlpSGD
117. mlpWeightDecay
118. mlpWeightDecayML
119. multinom
120. nb
121. nbDiscrete
122. nbSearch
123. neuralnet
124. nnet
125. nnls
126. nodeHarvest
127. oblique.tree
128. OneR
129. ordinalNet
130. ORFlog
131. ORFpls
132. ORFridge
133. ORFsvm
134. ownn
135. pam
136. parRF
137. PART
138. partDSA
139. pcaNNet
140. pcr
141. pda
142. pda2
143. penalized
144. PenalizedLDA
145. plr
146. pls
147. plsRglm
148. polr
149. ppr
150. protoclass
151. pythonKnnReg
152. qda
153. QdaCov
154. qrf
155. qrnn
156. randomGLM
157. ranger
158. rbf
159. rbfDDA
160. Rborist
161. rda
162. relaxo
163. rf
164. rFerns
165. RFlda
166. rfRules
167. ridge
168. rlda
169. rlm
170. rmda
171. rocc
172. rotationForest
173. rotationForestCp
174. rpart
175. rpart1SE
176. rpart2
177. rpartCost
178. rpartScore
179. rqlasso
180. rqnc
181. RRF
182. RRFglobal
183. rrlda
184. RSimca
185. rvmLinear
186. rvmPoly
187. rvmRadial
188. SBC
189. sda
190. sddaLDA
191. sddaQDA
192. sdwd
193. simpls
194. SLAVE
195. slda
196. smda
197. snn
198. sparseLDA
199. spikeslab
200. spls
201. stepLDA
202. stepQDA
203. superpc
204. svmBoundrangeString
205. svmExpoString
206. svmLinear
207. svmLinear2
208. svmLinearWeights
209. svmPoly
210. svmRadial
211. svmRadialCost
212. svmRadialSigma
213. svmRadialWeights
214. svmSpectrumString
215. tan
216. tanSearch
217. treebag
218. vbmpRadial
219. vglmAdjCat
220. vglmContRatio
221. vglmCumulative
222. widekernelpls
223. WM
224. wsrf
225. xgbLinear
226. xgbTree
227. xyf


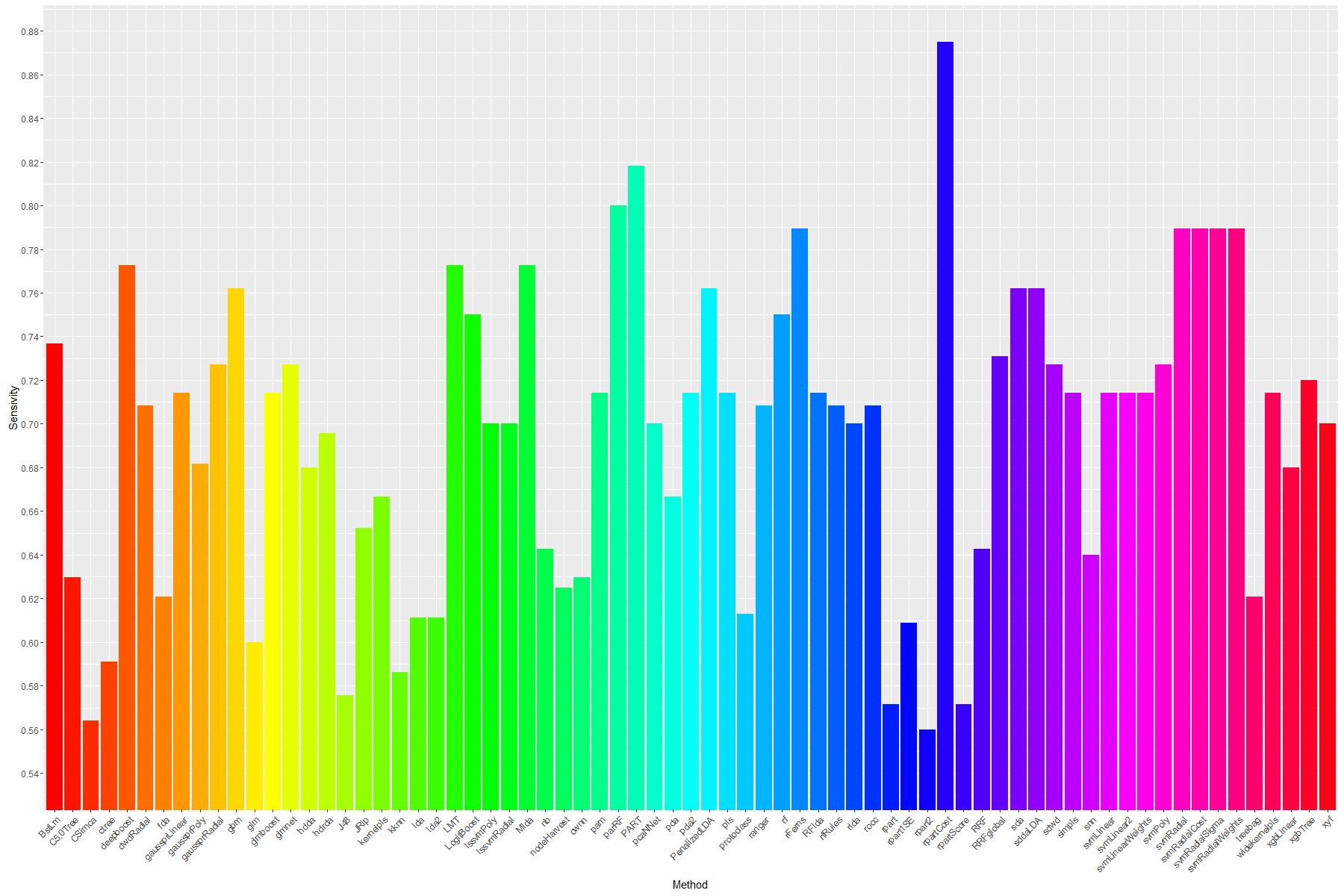


Figure S1: The sensitivity of all 227 classifiers with accuracy >50% in the independent test set.


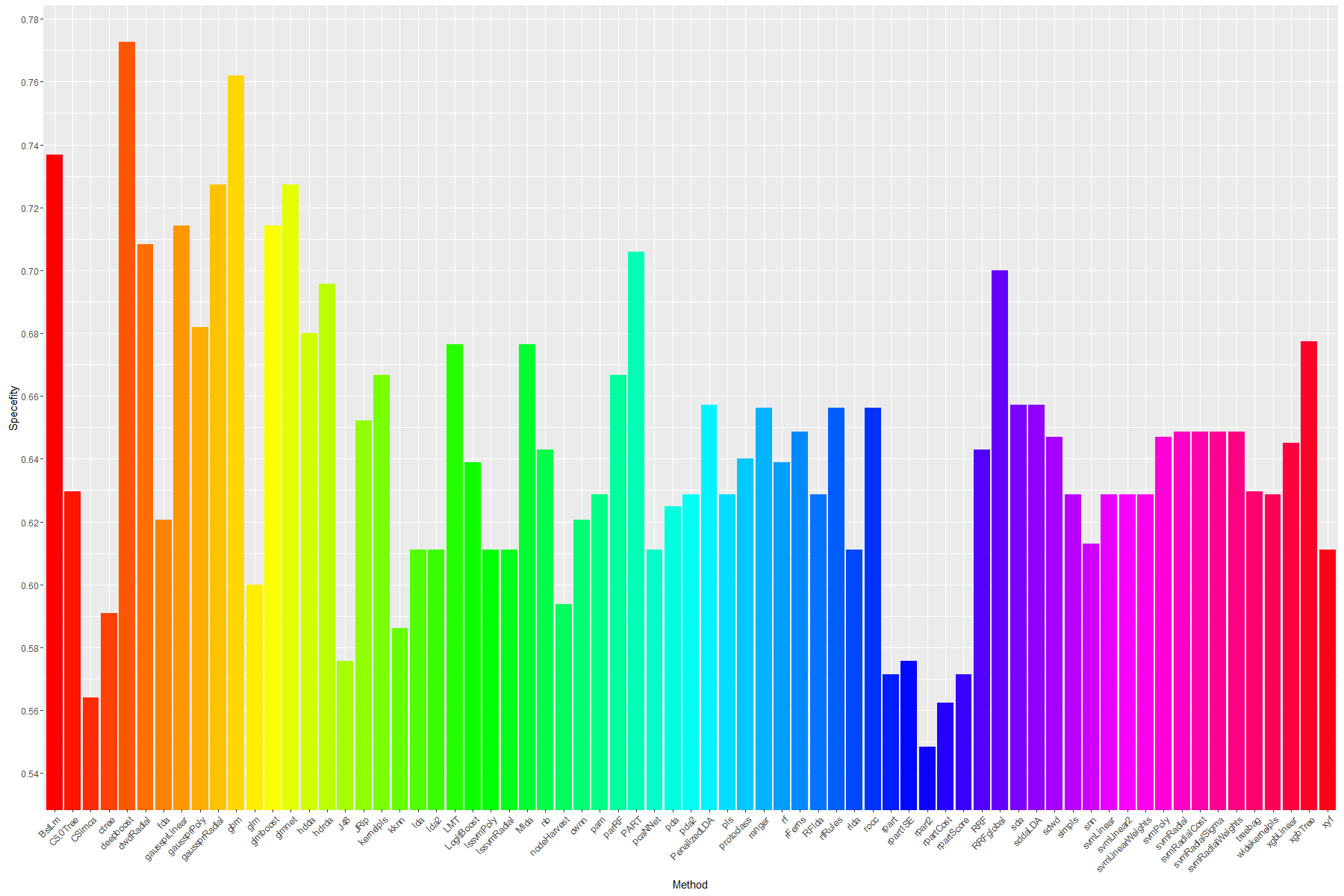


Figure S2: The specificity of all 227 classifiers with accuracy >50% in the independent test set.


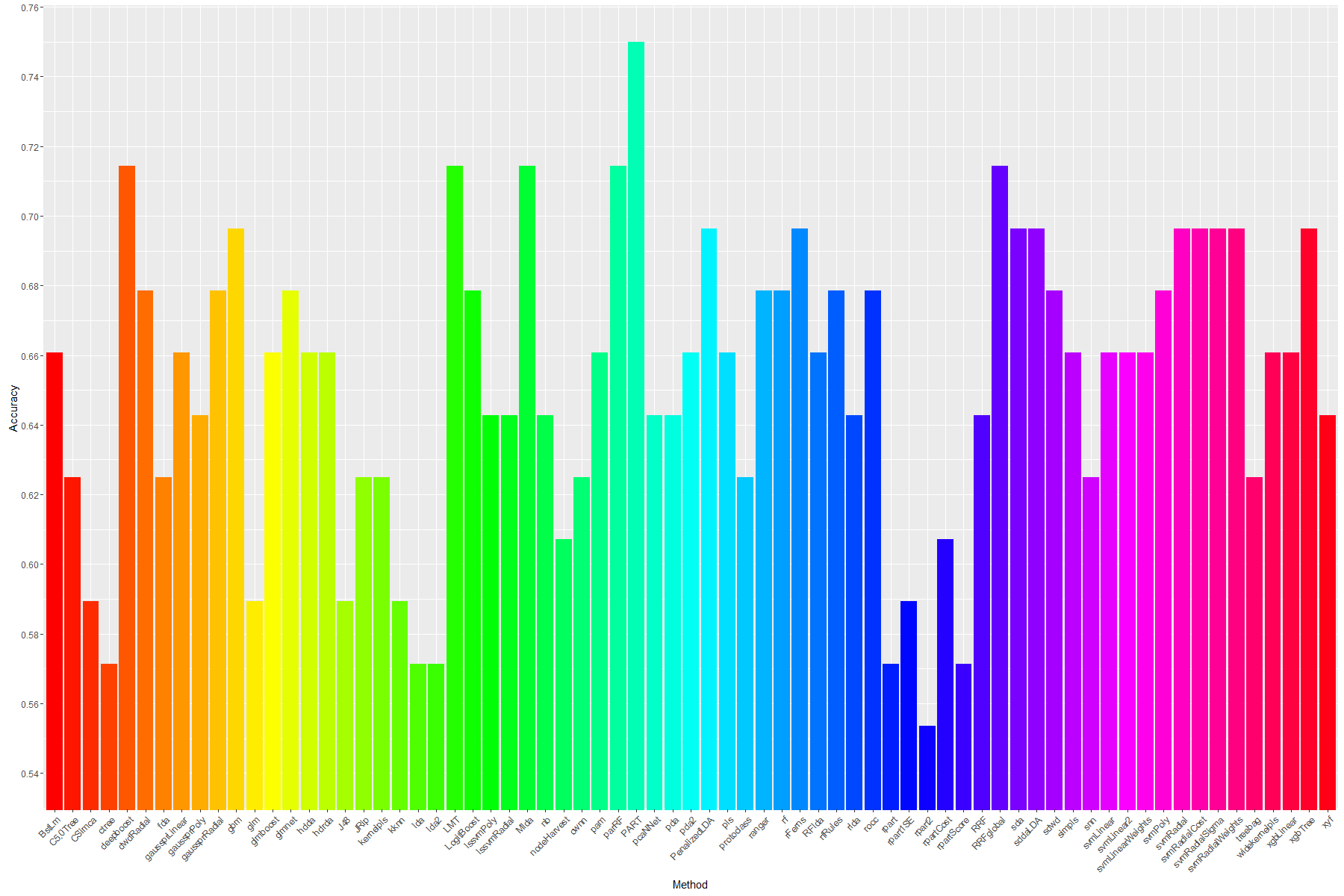


Figure S3: The accuracy of classifiers with accuracy >50% in the independent test set.
